# Detection and quantification of beef and pork materials in meat products by duplex droplet digital PCR

**DOI:** 10.1371/journal.pone.0181949

**Published:** 2017-08-03

**Authors:** Yicun Cai, Yuping He, Rong Lv, Hongchao Chen, Qiang Wang, Liangwen Pan

**Affiliations:** Technical Center for Animal, Plant and Food Inspection and Quarantine, Shanghai Entry-Exit Inspection and Quarantine Bureau of China, Pudong New Area, Shanghai, China; Wageningen UR Livestock Research, NETHERLANDS

## Abstract

Meat products often consist of meat from multiple animal species, and inaccurate food product adulteration and mislabeling can negatively affect consumers. Therefore, a cost-effective and reliable method for identification and quantification of animal species in meat products is required. In this study, we developed a duplex droplet digital PCR (dddPCR) detection and quantification system to simultaneously identify and quantify the source of meat in samples containing a mixture of beef (*Bos taurus*) and pork (*Sus scrofa*) in a single digital PCR reaction tube. Mixed meat samples of known composition were used to test the accuracy and applicability of this method. The limit of detection (LOD) and the limit of quantification (LOQ) of this detection and quantification system were also identified. We conclude that our dddPCR detection and quantification system is suitable for quality control and routine analyses of meat products.

## 1. Introduction

The food safety standards of the European Union require that meat products be labeled with accurate and detailed information, including the composition and percentage of meat from different source species [1]. However, since consumers have difficulty identifying the authenticity of some meat products, adulterated meat products are difficult to avoid. In 2013, adulterated horse meat [2, 3] and halal beef burgers adulterated with pork were discovered in some European countries. Incidents of adulteration increase consumer health and safety risks; therefore, accurate methods for detection and quantification of adulterated meat products are required.

There are several routinely used methods for the detection and quantification of adulterated meat products [4–6]. For the detection of species-specific proteins [7], both ELISA [8] and ultra-performance liquid chromatography [4, 9] are effective. For detection and quantification of nucleic acids, polymerase chain reaction (PCR) systems including conventional PCR [10, 11], quantitative polymerase chain reaction (qPCR) [12, 13], LAMP method [14], and droplet digital PCR (ddPCR) [15–17] have been used. Because of the high sensitivity and specificity, mitochondrial DNA (mtDNA) [18–20] is commonly used for species identification. However, mtDNA is not applicable for quantification purposes, because there are significant variations in mtDNA levels among species. Therefore, nuclear DNA is the optimal choice quantification of different species present in meat products [15].

The application of ddPCR for identification and quantification of plant and animal species has gained considerable interest in recent years, as ddPCR is able to quantify specific DNA sequences from as low as a single molecule template. Briefly, sample DNA is partitioned into thousands of nanoliter-sized droplet reaction bubbles by the Droplet Generator. Subsequently, specific DNA targets are amplified on a thermocycler, and bubbles that are positive or negative for fluorescent signal are counted and recorded by the Droplet Reader. Each individual droplet is defined as positive or negative based on its recorded fluorescence signal. Following the limiting dilution principle and using Poisson algorithms, this method is able to quantify the absolute count of a specific target DNA molecule in a sample [21]. Unlike traditional qPCR, ddPCR provides an absolute measure of nucleic acid copy number directly without transformation through any other intermediary value like cycle threshold (Ct) or fitting to a standard curve [22]. The microtiterization step separates each sample into thousands of droplets, which minimizes the deleterious effect of factors that can inhibit gene amplification and detection of the fluorescent signal, thereby increasing the overall accuracy and precision of target sequence quantification for each sample [22]. This direct approach for detection and quantification of specific DNA targets suggests that ddPCR may provide more precise and reliable quantification data, especially when applied to samples with mixed or complex makeups.

In this study, we established a dddPCR method to detect and quantify beef and pork components in meat products. This technique is simultaneously able to specifically identify and quantify beef and pork materials from meat products, and can be adapted for routine use in the quality control of meats and in detecting and preventing meat adulteration.

## 2. Materials and methods

### 2.1 Test material preparation

Fresh lean beef (*Bos taurus*), pork (*Sus scrofa*), chicken (*Gallus gallus*), and mutton (*Capra hircus*) were purchased from a local market in Shanghai, China. All the samples were minced, dried in a baking oven (UFE500AO, Memeert, Germany) at 80°C for 72 h, and ground into a superfine powder in liquid nitrogen using a bench-top 6850 Freezer/Mill set at 10 Hz for 8 min (SPEX SamplePrep, Metuchen, NJ) [16]. Mixed beef and pork powder samples of known proportion, ranging from 5%–to 95% beef/pork by mass (95 mg _beef_/ 5 mg _pork_, 80 mg _beef_/ 20 mg _pork_, 75 mg _beef_/ 25 mg _pork_, 60 mg _beef_/ 40 mg _pork_, 55 mg _beef_/ 45 mg _pork_, 40 mg _beef_/ 60 mg _pork_, 35 mg _beef_/ 65 mg _pork_, 20 mg _beef_/ 80 mg _pork_, 15 mg _beef_/ 85 mg _pork_, 10 mg _beef_/ 90 mg _pork_), were prepared and used to assess the validity and sensitivity of the method. These powders were used as a reference material for DNA extraction, quantification, and for testing the fluorescence interference in the dddPCR system. In order to guarantee that the extracted DNA accurately represents the proportion of different meats within a sample, the Freezer Mixer was used to grind the mixtures evenly to ensure complete mixing. Meat samples from individual species or mixed meat samples were prepared in a total mass of 100 g. For each sample, the total mass of 100 g was prepared and DNA was extracted from 100 mg of the sample.

To verify the species specificity of the assays, reference DNA samples from 20 different species were purchased from Zyagen Laboratories (San Diego, CA, USA). All reference DNA samples were verified by sequencing and specific sequence alignment [23].

### 2.2 Primers and probes

For the detection and quantification of beef (*Bos taurus*) and pork (*Sus scrofa*), the *Bos taurus* beta-actin (ACTB) gene (GenBank accession number: EH170825) [24] and *Sus scrofa* ACTB gene (GenBank accession number: DQ452569) [25] were selected as the target detection sequences. Primers and probes were designed using Primer Express Software version 3.0 (Applied Biosystems, Foster City, CA, USA) and purchased from Invitrogen (Thermo Fisher Scientific, USA). Primers and probes were subjected to specificity and homology analyses by BLAST searches against the entire GenBank database. The nucleotide sequences of the primers and probes were designed to meet optimal conditions for ddPCR. The FAM fluorophore and minor groove binder (MGB) were used for detection of bovine sequences. A probe labeled with VIC fluorophore (Thermo Fisher Scientific, USA) and MGB quencher was used for detection of porcine sequences (Table 1).

**Table 1:** Primer and probe sequences for multiplex dPCR experiment.

Primer/Probe	Sequence/labeling	GenBank accession number
Bos-ACTB-63bp-F	GCGGCCTCGGAGTGTGTA	Beta-actin gene
Bos-ACTB-63bp-R	CCCCAGAATGAGGTTCACTTCA	EH170825
Bos-ACTB-63bp-P	FAM-TCAGTAGGTGCACAGTAC-MGB	
Sus-ACTB-97bp-F	CGTAGGTGCACAGTAGGTCTGAC	Beta-actin gene
Sus-ACTB-97bp-R	GGCCAGACTGGGGACATG	DQ452569
Sus-ACTB-97bp-P	VIC-CCAGGTCGGGGAGTC-MGB[16]	

### 2.3 DNA extraction

DNA was extracted from meat samples by phenol/chloroform extraction [16]. All samples (100 mg) were mixed with 800 μl histiocyte lysis buffer (TIANGEN Biotech, Beijing, China) and 100 μg proteinase K (TIANGEN Biotech, Beijing, China). Following 60 min incubation at 65°C with occasional vigorous shaking, an equal volume of phenol/chloroform was added; samples were mixed and centrifuged at 12,000 rpm for 10 min. The resulting supernatant was collected, and an equal volume of chloroform was added; samples were mixed and centrifuged at 12,000 rpm for 5 min. The aqueous layer was transferred to a clean tube. An equal volume of ice-cold 100% EtOH and a one-tenth volume of 3M sodium acetate (pH 5.2) were added; samples were mixed incubated at -20°C for 30 min, and centrifuged at 12,000 rpm for 30 min. The supernatant was discarded and the pellet was washed twice with 800 μl 75% EtOH. Following centrifugation at 12,000 rpm for 5 min, the pellet was air-dried and resuspended in 100 μl DNAse-free and RNAse-free water (Invitrogen, Carlsbad, CA, USA). The DNA concentration of each sample was measured in a NanoVue spectrophotometer (GE Healthcare China, Beijing, China).

### 2.4 Specificity

To evaluate the species specificity of the assay, DNA from 20 different animal species was used as template for PCR reactions. The species evaluated are as follows: cow (*Bos taurus*), donkey (*Equus asinus*), sheep (*Ovisaries*), goat (*Capra hircus*), horse (*Equus caballus*), chicken (*Gallus gallus*), duck (*Anas platyrhynchos*), goose (*Anse ranser*), turkey (*Meleagris gallopavo*), pig (*Sus scrofa)*, quail (*Coturnix coturnix*), camel (*Camelus dromedarius*), dog (*Canis lupus familiaris*), ferret (*Mustela putorius furo*), rabbit (*Oryctolagus cuniculus*), pigeon (*Columba livia*), mouse (*Mus musculus*), rat (*Rat tusnorvegicus*), Rhesus monkey (*Macaca mulatta*), and carp (*Cyprinus carpio*).

### 2.5 Fluorescence interference

To assess fluorescence interference between the bovine and porcine specific probes, we evaluated the dddPCR system by using DNA derived from single-species samples or combined-species samples (beef, pork, chicken, and mutton). The deviation of the measured value relative to the true value was calculated from three measurements. Three independent experiments were performed.

### 2.6 Droplet digital PCR assay

The 20 μl ddPCR reaction mixture consisted of 1.8 μl of forward and reverse primers (final concentration, 900 nM), 0.5 μl of the probe (final concentration, 250 nM), 10 μl of ddPCR Master Mix (Bio-Rad, Hercules, CA, USA), 4 μl of template DNA (40 × diluted), and 1.9 μl of nuclease- and protease-free water (Thermo Scientific, Salt Lake City, UT, USA).

The 20 μl dddPCR reaction mixture consisted of 0.9 μl of each of the four primers (final concentration, 900 nM), 0.5 μl of each of the two probes (final concentration, 250 nM), 10 μl of ddPCR Master Mix (Bio-Rad, Hercules, CA, USA), 4 μl of template DNA (40 × diluted), and 1.4 μl of nuclease- and protease-free water (Thermo Scientific, Salt Lake City, UT, USA).

The reaction mixture was divided into approximately 20,000 droplets using a QX200 ddPCR droplet generator (Bio-Rad, Hercules, CA, USA). The target DNA segments and ddPCR reagents were randomly dispersed among the ~20,000 droplets. Conventional PCR was performed in a T100^™^ Thermal Cycler (Bio-Rad, Hercules, CA, USA) according to the following cycling protocol: one enzyme inactivation cycle at 95°C for 10 min, followed by 40 cycles of denaturation at 94°C for 30 s and annealing/extension at 60°C for 1 min, followed by one enzyme inactivation cycle at 98°C for 10 min. Finally, the reaction mixture was held at 4°C. Following droplet reading, analyses, and calculations, DNA concentration was determined by Poisson distribution analysis.

### 2.7 Limit of detection (LOD) and Limit of quantitation (LOQ)

Whole-genome DNA from beef and pork was used to determine the LOD and the LOQ of the dddPCR detection system. A serial dilution series of the DNA solution containing a mixture of bovine and porcine DNA was produced: 10, 5, 2.5, 1, 0.5, 0.1, 0.05, 0.01 and 0.005 ng/μl. To produce the dilution series, 0.2 × TE buffer was used as the dilution buffer. For each dilution, 12 replicates were evaluated and three independent experiments were carried out. The DNA concentration was measured spectrophotometrically (NanoVue, GE Healthcare China, Beijing, China).

### 2.8 Linear equation and the derivation of the formula

Powdered samples of beef and pork were accurately weighed (5, 10, 20, 30, 40, 50, 60, 70, 80, 90, 100 and 120 mg, with n = 3 each) in a precision electronic balance (Sartorius China, Beijing, China). Whole-genome DNA was extracted and the nucleic acid concentration was measured spectrophotometrically (NanoVue, GE Healthcare China, Beijing, China). A relationship between the meat powder weight and the corresponding nucleic acid content was established. In order to establish the relationship between nucleic acid content (ng) and target DNA copy number, the serially diluted meat DNA and non-template control (NTC) DNA samples (5, 10, 20, 40, 60, 80, 100, 120, 140 and 160 ng/μl, with n = 3 each) were analyzed by ddPCR. For each test, three independent experiments were performed.

The nucleic acid content of a sample has a direct relationship with meat weight, as defined by two linear equations: the first linear equation describes the relationship between specific meat powder weight and meat DNA concentration; the second linear equation describes the relationship between DNA copy number and the meat DNA weight. Considering meat DNA weight as an intermediate parameter, through mathematical derivation procedures, we can determine the equation for calculating the original raw meat weight from the specific DNA copy number.

### 2.9 Analysis of samples of known concentration

The presence of more than two types of animal meat in food or feed products is common. DNA from these food products can be used for identification and quantification. However, some factors may affect DNA extraction and detection, including meat species, production processes, DNA degradation, tissue composition, and amplification efficiency. To assess the accuracy and applicability of dddPCR, ten meat samples of known composition were prepared and analyzed.

## 3. Results and discussion

### 3.1 Specificity

The specificity of this dddPCR detection system was evaluated for the 20 species listed above. Bovine and porcine primer/probe combinations were evaluated in this dddPCR detection system to assess the presence of cross reactions with other species. No cross reactions or cross amplifications were observed, indicating that the primer/probe combinations used in this study can be used to specifically and reliably identify and quantify beef and pork materials in mixed meat products.

### 3.2 Fluorescence interference

**Fig 1:**
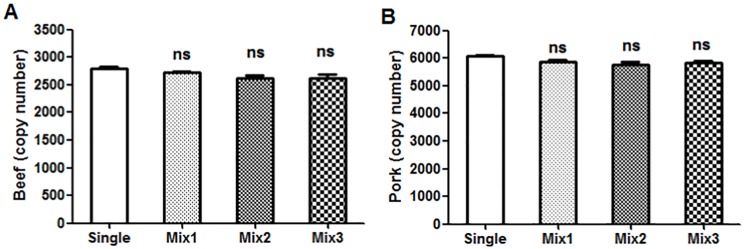
Single and mixed animal DNA samples for the fluorescence interference test. Single samples contained beef or pork DNA. Mixed samples included Mix1 (100 ng beef and 100 ng pork DNA), Mix2 (100 ng beef, 100 ng pork, and 100 ng chicken DNA), and Mix3 (100 ng beef, 100 ng pork, 100 ng chicken, and 100 ng mutton DNA). Three replicates per sample were analyzed by dddPCR. The values are expressed as mean ± SEM. Each value represents the average of three experiments. Error bars represent the standard deviation between the three replicates. ns: non-significant difference compared to the single group.

Fluorescence interference occurs when multiple fluorescence reporters (in this case, FAM and VIC) are amplified and detected in the same reaction well at the same time. This fluorescence interference phenomenon can directly affect the accuracy of the detection results. In order to evaluate if this phenomenon occurs in our dddPCR method, DNA was isolated from beef, pork, chicken, and mutton. Following purification and quantification, DNA samples from single-species meat sources or from meat sources combined from multiple species were evaluated by dddPCR. The different samples were as follows: Mix1 (beef and pork), Mix2 (beef, pork and chicken), and Mix3 (beef, pork, chicken, and mutton). The DNA content from each species was 100 ng. There were no significant differences in DNA copy number between samples of beef alone (Fig 1A) or pork alone (Fig 1B) and the mixed samples (Mix1, Mix2, and Mix3. We did not observe fluorescence interference phenomenon in any samples containing DNA from a multiple animal species; this indicated the high specificity of the primers and probes. These data demonstrate that these two fluorescence reporters can be used together without interference in our dddPCR detection system.

### 3.3 LOD and LOQ

LOD in this study was defined as the smallest concentration at which all sample replicates gave a positive qualitative result, indicating that the detection rate should be above 95%. In three independent experiment the dddPCR detection method demonstrated good assay performance and high sensitivity (Table 2). The LOD was determined to be as low as ~0.1 ng/μl for beef and pork.

**Table 2:** Limit of detection for beef and pork DNA in dddPCR assays (n = 36).

ng/μl	ng/tube	BP	BN	DR	PP	PN	DR
**10**	40	36	0	100%	36	0	100%
**5**	20	36	0	100%	36	0	100%
**2.5**	10	36	0	100%	36	0	100%
**1**	4	36	0	100%	36	0	100%
**0.5**	2	36	0	100%	36	0	100%
**0.1**	0.4	36	0	**100%**	35	1	**97.20%**
**0.05**	0.2	28	8	77.80%	24	12	66.70%
**0.01**	0.04	18	18	50%	0	36	0%
**0.005**	0.02	0	36	0%	0	36	0%

BP = Beef Positive, BN = Beef Negative, PP = Pork Positive, PN = Pork Negative, DR = Detection Rate.

Based on the “*FAO Guidelines on performance criteria and validation of methods for detection*, *identification and quantification of specific DNA sequences and specific proteins in food*” [15], the LOQ of the dddPCR system was defined as the lowest concentration with a coefficient of variation (CV) ≤ 25% for quantification. A standard curve was generated from samples of known DNA concentration, and the CV of adjacent points was calculated (Table 3). The off-limits CV values for this detection system for beef and pork were 47.08% and 30.16%, respectively. Both off-limits CV values appeared in the 0.5 ng/μl level, which is an order of magnitude higher than the LOD for this detection system, indicating that the LOQ of this detection system is around 0.5 ng/μl level for both beef and pork.

**Table 3:** Limit of quantification for beef and pork DNA in dddPCR assays (n = 36).

	Copy number		Coefficient of variation	
ng/μl	Beef	Pork	Beef	Pork
**10**	4601	3043	NC	NC
**5**	2231	1541	-3.12%	1.31%
**2.5**	1082	799	-3.13%	3.54%
**1**	409	321	-5.72%	0.47%
**0.5**	229	156	10.83%	-3.12%
**0.1**	87	45	**47.08%**	**30.16%**
**0.05**	44	23	1.37%	1.82%
**0.01**	11	5	22.04%	11.88%
**0.005**	ND	ND	NC	NC

ND = not detected, NC = not calculated

### 3.4 DNA extraction efficiency

**Fig 2:**
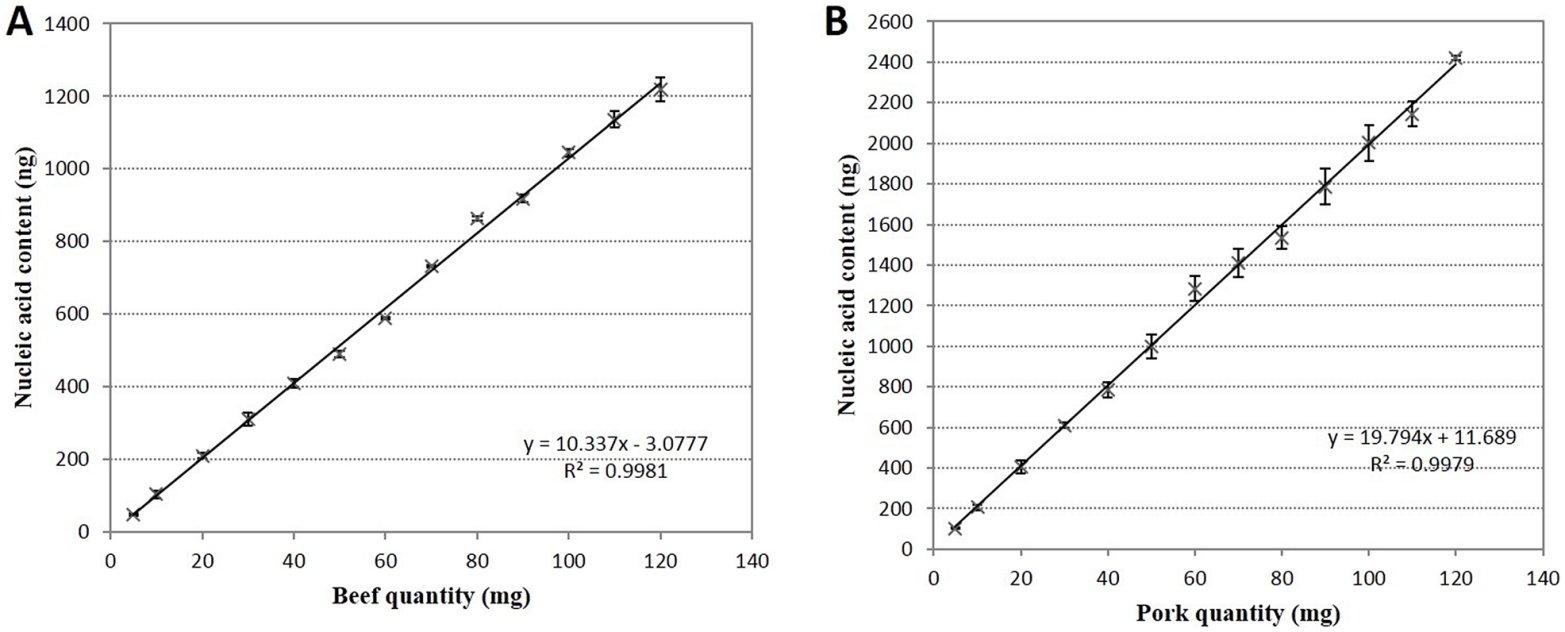
Relationship between meat quantity (mg) and nucleic acid content (ng). After accurate weighing and DNA extraction, the nucleic acid (ng) content of three replicates of each sample was recorded. The DNA extraction efficiency was different between beef samples and pork samples. Within the 5–120 mg weight range, the initial sample weight (mg) and nucleic acid (ng) content have a linear relationship: R^2^ = 0.9981 for beef (A) and R^2^ = 0.9979 for pork (B).

To establish a linear relationship between meat mass and nucleic acid content, DNA was harvested form meat samples of known mass by manual phenol/chloroform extraction. Extraction efficiency can be impacted by the presence of different tissue types (fat, skin, internal organs) and complex meat composition, therefore we used fresh lean meat samples consisting primarily of muscle tissue. DNA was extracted and measured from samples of beef (5–120 mg), pork (5–120 mg), and non-targeting species control (NTC) [16]. The results revealed a linear relationship between beef powder mass and nucleic acid content (*R*^*2*^ = 0.998; Fig 2A). Similarly, a linear relationship was identified between pork powder mass and nucleic acid content (*R*^*2*^ = 0.997; Fig 2B). We observed that there were differences in the slope and intercept values between the equation obtained in this study and those obtained in a previous study [16], which could be attributed to the meat samples used and experimental error in the ddPCR system.

### 3.5 DNA detection by ddPCR

**Fig 3:**
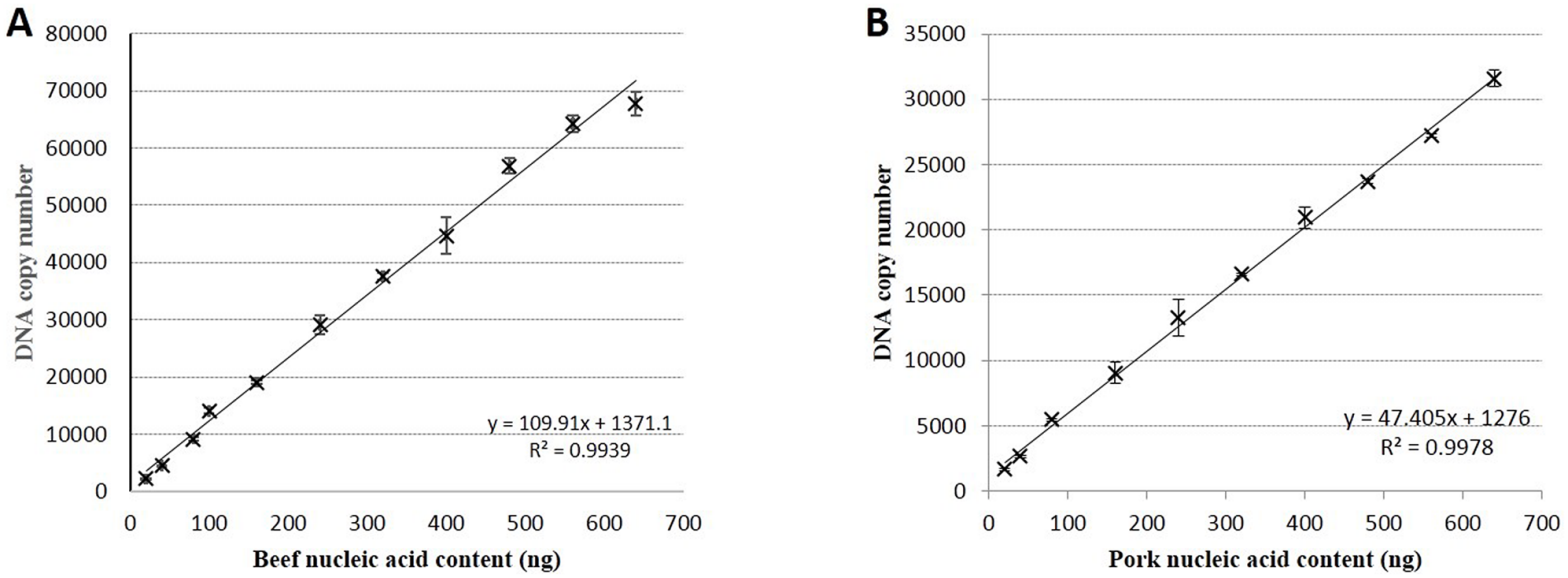
Relationship between nucleic acid content (ng) and target DNA copy number. Beef and pork DNA samples of known concentrations were prepared and analyzed by ddPCR. Each detection point data is the average of triplicate samples from three independent experiments. Sample nucleic acid (ng) content and DNA copy number have a linear relationship: R^2^ = 0.9939 for beef (A) and R^2^ = 0.9978 for pork (B).

To identify the linear relationship between total DNA weight and target specific DNA copy number, serially diluted DNA extracted from beef and pork meat samples were analyzed by ddPCR; non-targeting species DNA was used as a control. Three replicates per diluted DNA sample were analyzed in three independent experiments. The total DNA mass and specific target DNA copy number demonstrated a linear relationship (*R*^*2*^ = 0.9939 and *R*^*2*^ = 0.9978 in beef and pork samples, respectively; Fig 3A and 3B).

### 3.6 Calculation of mass of beef and pork based on DNA quantification

Equations for calculating the weight of meat based on the DNA quantification were established. The quantity of meat in beef samples can be determined based on the linear relationship between meat quantity (mg) and nucleic acid content (ng) (Y_1_ = 10.33*X_1_-3.1; X_1_ represents the DNA concentration in ng/μl; Y_1_ represents the weight of the specific meat powder in mg; Fig 1A). Alternatively, the quantity of meat in beef samples can be determined based on the DNA copy number in a sample, as we established a linear relationship between nucleic acid content (ng) and DNA copy number (Y_2_ = 109.9*X_2_+1371.1; X_2_ represent the DNA mass in ng; Y_2_ represents the DNA copy number; Fig 3A). In the ddPCR experiments, due to the limit of the instrument’s detection range, DNA samples should be diluted at least 40-fold to ensure that the upper limit of detection is not exceeded. The final quantity of dilute sample per ddPCR reaction was 4 μl/tube in a final volume of 20 μl/tube; from this we derive the relationship (X_2_/4)*40 = Y_1_. The two former parameters, X1 and Y2, can be brought together into one equation: (Y_2_-1371)*10/110 = 10.33*X1-3.077. To facilitate visualization and understanding, copy number concentration (copies/μl) can be represented by C_beef_, and can be directly determined from the ddPCR test. “M” represents the weight of specific meat powder in mg. Therefore, Y_2_ = 20 C_beef_ and M = X_1_. These parameters can be brought into a single equation: (20C_beef_-1371)*10/110 = 10.33M-3.077. The final equation to determine mass of beef based on DNA copy number is: M_beef_ = 0.17C_beef_-11.8. Similarly, the equation to determine pork mass based on DNA copy number was derived: M_pork_ = 0.21C_pork_-14.2.

### 3.7 Analyses of samples of known concentration

**Fig 4:**
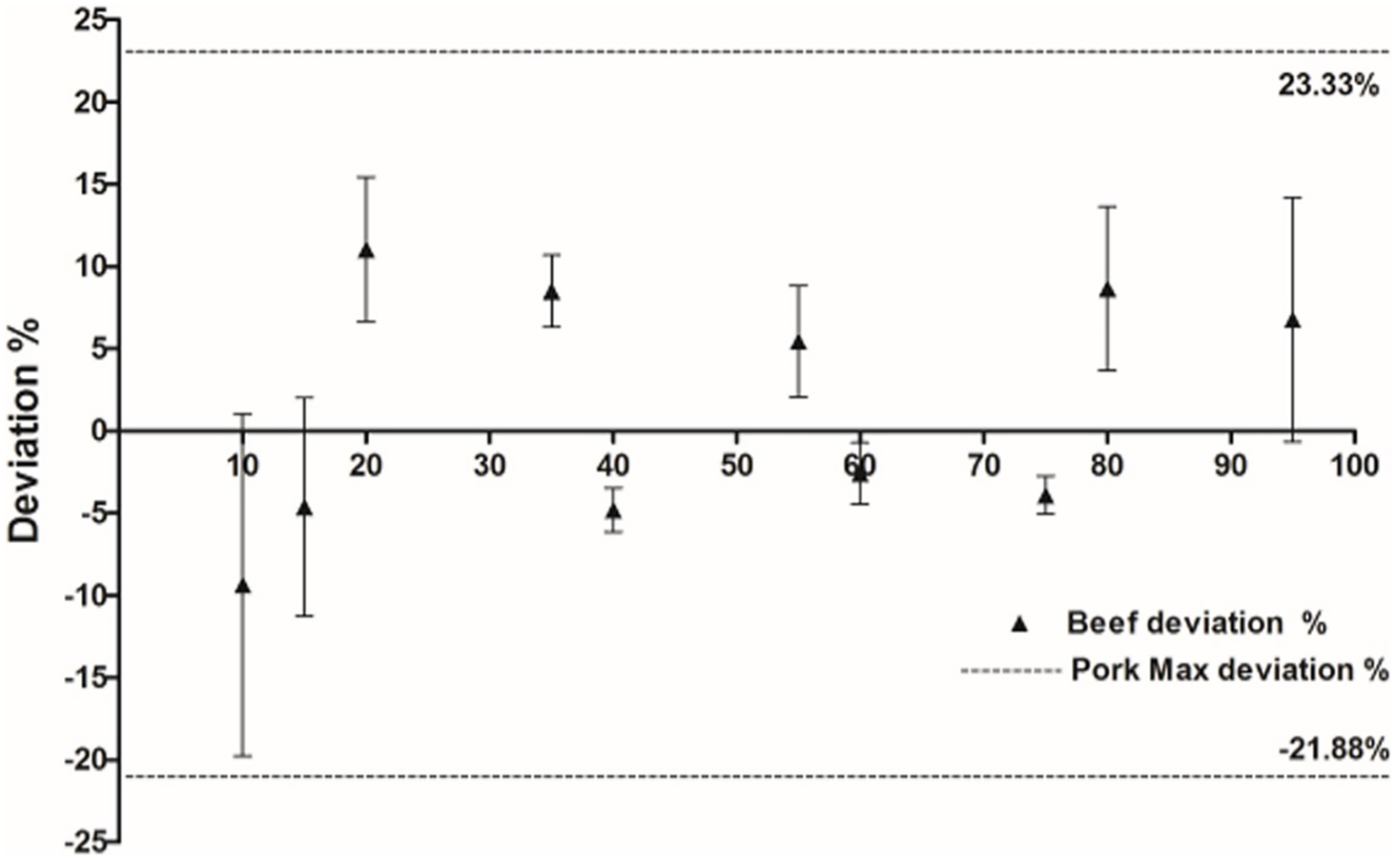
Deviation of the beef and pork samples detected by multiplex ddPCR. The deviation of the beef samples is indicated by a triangle. The maximum deviation of the pork samples (+23.33% and -21.88%) is indicated by a dotted line. Error bars represent the standard deviation of the beef deviation tested by ddPCR at each concentration (three replicates per concentration).

DNA was extracted from mixed samples of beef and pork in triplicate, in the following beef: pork ratios: 95:5; 80:20; 75:25; 60:40; 55:45; 40:60; 65:35; 20:80; 15:85; 10:90. Single and mixed DNA samples were diluted 40-fold, and 4 μl of each sample was evaluated by ddPCR using species-specific primers and the FAM and VIC fluorescent probes in one ddPCR reaction tube. Following droplet generation, PCR amplification, and fluorescence detection, the copy number of bovine and porcine DNA was determined and the weight of each single component (beef or pork) in the mixed sample was calculated; the data are summarized in Table 4. Compared to the true ratio of the ten pre-made samples, the measured value of beef and pork mass in the samples was not significantly different. Additionally, the dddPCR quantification system demonstrated consistency and reproducibility, based on the data obtained from triplicate experiments. To assess accuracy, the deviation between the observed and real meat weight were calculated. The deviation of beef varied between -9.37% and 11.03%. For pork, the deviation varied from -21.88% to 23.33%. Pork samples showed higher deviation than beef samples in this experiment (Fig 4), which could be attributed to heterogeneity in lean and fat meat content.

**Table 4:** Quantification results of samples with known concentrations.

	Beef			Pork		
	True (mg)	Measured (mg)	Deviation	True (mg)	Measured (mg)	Deviation
**Sample 1**	95.00	101.42	6.76%	5.00	6.17	23.33%
**Sample 2**	80.00	86.93	8.66%	20.00	15.62	-21.88%
**Sample 3**	75.00	72.08	-3.90%	25.00	28.35	13.39%
**Sample 4**	60.00	58.45	-2.58%	40.00	43.03	7.58%
**Sample 5**	55.00	58.00	5.45%	45.00	47.09	4.64%
**Sample 6**	40.00	38.08	-4.81%	60.00	66.85	11.42%
**Sample 7**	35.00	37.97	8.50%	65.00	68.82	5.88%
**Sample 8**	20.00	22.21	11.03%	80.00	83.01	3.77%
**Sample 9**	15.00	14.31	-4.62%	85.00	93.04	9.45%
**Sample 10**	10.00	9.06	-9.37%	90.00	89.12	-0.98%

## 4. Conclusions

In recent years, increases in the incidence of adulteration and mislabeling of animal products has raised significant attention and concern among consumers and regulatory agencies. Adulteration of meat food products can have serious consequences, as it directly relates to people’s food safety and health. Due to the similar histological structure of different meat products, the common consumer is not able to readily distinguish or identify improperly labeled composition or proportion in mixed meat products. qPCR is the most commonly used method for quantification of animal materials in mixed meat products; it is recommended that standard curves be prepared and tested every time by using serial dilutions of DNA extracted from reference material. The quantification accuracy and efficiency of this procedure can be influenced by many factors, including amplification inhibitors and accuracy of the standard curve, leading to significant under- or over-estimation of the content of animal materials in food and meat products. Therefore, an accurate, effective, and convenient quantification method for animal materials in food and meat products is required.

Here we report the establishment of a novel dddPCR method for the simultaneous quantification of beef and pork materials in food and meat products. The beef and pork specific primer/probes tested in this paper demonstrate good specificity and sensitivity. For specificity, neither primer/probe combination showed cross-reactivity or nonspecific amplification. The sensitivity (LOD) of this dddPCR detection system is in about 0.1 ng/μl level. At the same time, fluorescence interference phenomenon was not observed in this dddPCR quantification system, regardless of the intricate background or complex nature of the samples. The LOQ of the beef and pork in dddPCR systems was estimated as the DNA concentration within the dynamic range and with a CV ≤ 25%. Based on this criterion, the LOQ was estimated to be around 0.5 ng/μl level for beef and pork (Table 3). Compared with experimental data from ddPCR studies [16], no significant variation of the species-specific target gene copy number, as measured by the dddPCR assays, was observed. We show that, based on the specificity, repeatability, consistency, limit of detection, and limit of quantification of the assay, the dddPCR assay has comparable detection performance to ddPCR. Based on the relationships we identified between DNA quantification and mass of beef or pork in a sample, the dddPCR system is ideally suited for development as a standard assay. In this system, no optimization of experimental condition or reagents is needed. The dddPCR assay has the advantages of reducing cost and time required for quantification of meat composition in complex samples. Similar experimental conclusions have been reported in other literatures [17].

For the practical implementation of the dddPCR approach as a standard assay, interlaboratory validation should be performed to demonstrate the robustness and reproducibility of the assay [26]. In conclusion, our dddPCR method demonstrated good performance in identifying the beef and pork content in mixed samples based on DNA content, indicating that this technique has the potential to facilitate screening for food adulteration and mislabeling in food and meat products.
